# P-2043. Outpatient Antiviral Treatment Among High-risk Patients with Mild to Moderate COVID-19 During the Omicron Surge

**DOI:** 10.1093/ofid/ofae631.2199

**Published:** 2025-01-29

**Authors:** Lingshan Syue, Nanyao Lee, Wen-Chien Ko

**Affiliations:** National Cheng Kung University Hospital, SFO, California; National Cheng Kung University Hospital, SFO, California; National Cheng Kung University Hospital, SFO, California

## Abstract

**Background:**

In response to the COVID-19 pandemic, outpatient treatment with oral antiviral medications has emerged as a crucial strategy to manage the disease effectively. This study aimed to evaluate the clinical outcomes of such treatment modalities in a cohort of confirmed COVID-19 patients.

**Methods:**

This is a retrospective study of COVID-19 cases with high risk diagnosed during an Omicron wave from May 2022 to December 2022 in Taiwan. Cases received either nirmatrelvir-ritonavir (NMV/r) or molnupiravir (MOV) as outpatient treatment were enrolled to assess the clinical effects of therapeutic strategies. The 30-day crude mortality and hospitalization were the primary endpoint.

**Results:**

Among 2932 patients with prescriptions of antiviral agents, 1395 patients received NMV/r, and 1537 received MOV. The average age of the patients was 63.8 years, with a majority (59%) being over 65 years old. Common comorbidities included diabetes (24.7%), chronic kidney disease (20.7%), cardiovascular diseases (17.6%), and a history of cancer (15.6%). The overall hospitalization rate was 15%, with a mortality rate of 0.7% which revealed promising clinical outcomes with outpatient treatment using either NMV/r or MOV. Compared with NMV/r, those on MOV were older (59.6 ± 18 years vs 67.4 ± 15 years, p< 0.001) and the proportion of cardiovascular disease (8.7% vs 19.5%) and chronic kidney disease (4.1% vs 28.5%) were higher. COVID-related 30-day hospitalization rate and mortality were higher in the MOV group significantly (15.5% vs 8.5%, p < 0.001; 0.8% vs 0.4%, p< 0.0001).

**Conclusion:**

Outpatient treatment with NMV/r and MOV demonstrated effectiveness in reducing hospitalization and mortality rates among COVID-19 patients. Therefore, selecting outpatient treatment with these antiviral medications could represent an effective strategy for managing COVID-19, contributing to improved patient outcomes and reduced hospitalization needs.

**Disclosures:**

All Authors: No reported disclosures

